# Silencing of LncRNA KCNQ1OT1 confers an inhibitory effect on renal fibrosis through repressing miR-124-3p activity

**DOI:** 10.1080/21655979.2022.2056816

**Published:** 2022-04-21

**Authors:** Jian Hao, Yun Zhou, Weimin Yu, Hui Li, Dandan He

**Affiliations:** a Department of Nephrology, The Fifth Clinical Medical College of Shanxi Medical University, Taiyuan, Shanxi Province, China; b Department of Nephrology, Shanxi Bethune Hospital, Taiyuan, Shanxi Province, China

**Keywords:** LncRNA KCNQ1OT1, MiR-124-3p, proliferation, extracellular matrix, renal fibrosis

## Abstract

LncRNA have been increasingly shown that plays pivotal roles in the development of various diseases, including renal fibrosis. Nevertheless, the pathological function of Long non-coding RNA KCNQ1OT1 (KCNQ1OT1) in the renal fibrosis remains obscure. Unilateral ureteral obstruction (UUO) was used to induce renal fibrosis. We detected the expression levels of KCNQ1OT1 in the TGF-β1-induced HK-2 cells via RT-qPCR analysis. The functions of KCNQ1OT1 on the progression of renal fibrosis were examined by CCK-8, EdU, dual-luciferase reporter, and immunofluorescence analyses. In the present study, we found that sh-KCNQ1OT1 obviously attenuated UUO-induced renal fibrosis. Moreover, production of extracellular matrix (ECM), including α-SMA and Fibronectin levels, was significantly increased in kidney and HK-2 cells after UUO or TGF-β stimulation. Knockdown of KCNQ1OT1 inhibited cell proliferation and inhibits the α-SMA and Fibronectin expression of TGF-β1-induced HK-2 cells. In addition, bioinformatics analysis and dual-luciferase reporter assay indicated that miR-124-3p was a target gene of KCNQ1OT1. Mechanistically, silencing miR-124-3p abolished the repressive effects of KCNQ1OT1 on TGF-β1-induced HK-2 cells. In conclusion, KCNQ1OT1 knockdown plays an anti-fibrotic effect through promotion of miR-124-3p expression in renal fibrosis, which provides a promising therapeutic target for the treatment of renal fibrosis.

## Graphical Abstract

**Figure:**
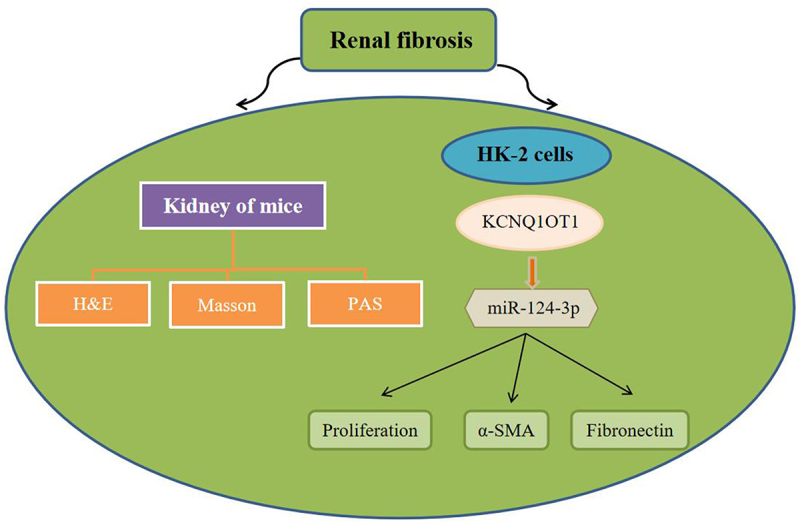

## Highlights

- The pathological function of KCNQ1OT1 in the renal fibrosis was explored.
- KCNQ1OT1 negatively modulated miR-124-3p.
- Elevated miR-124-3p reversed the influence of KCNQ1OT1.
- KCNQ1OT1 knockdown plays an anti-fibrotic effect by targeting miR-124-3p in renal fibrosis.

## Introduction

Chronic kidney disease (CKD) is a type of progressive diseases and a critical global public health issue that leading contributions to death globally with a prevalence rate of 10% to 12% in the world [1–4]. Owing to lack of renal reserve function and clinical features in the early onset, CKD generally progresses to the middle and late stages when obvious symptoms were appeared [5]. Fibrosis is a hallmark and common pathway leading to end-stage organ dysfunction [6]. Renal interstitial fibrosis, as the final pathological hallmark of CKD, irreversibly damages of renal function, and the final convergent pathway of renal disease [7–9].

Renal fibrosis is the usually ultimate manifestation of various kidney diseases including glomerulonephritis and diabetic nephropathy [10]. In histological features, renal fibrosis manifests the excessive deposition of extracellular matrix (ECM) [11], which is always accompanied by various pathological alterations in tubular epithelial cells including epithelial-to-mesenchymal transition (EMT), fibroblast activation, immune cell infiltration, and apoptosis [12,13].

Despite that extensive research exists on the pathogenesis and molecular mechanisms underlying of renal fibrosis, the effective intervention for this disease is still lacking. In view of that renal fibrosis can lead to scar formation and even renal failure, further exploring the novel mechanisms of renal fibrosis, as well as searching for novel CKD therapeutic targets is compellingly needed, which is of great significance for the development of effective therapeutic strategies and prevention of patients with renal diseases.

Long non-coding RNAs (lncRNAs) are a type of RNAs whose transcripts are more than 200 nucleotides in length, and have no/little potentials in coding proteins [14,15]. A growing body of evidence indicates that lncRNAs play extensive regulatory roles in life activities and the development of many diseases and exert vital roles in the regulation of cell proliferation, migration, invasion, and apoptosis [16–19]. In recent years, researchers have proved lncRNAs act as important regulators in the progress of fibrosis. For instance, silencing lnc-Hser aggravated liver fibrosis through inducing the epithelial–mesenchymal transition (EMT) and apoptosis [20]. Liu et al. proved that lnc-PCF can accelerate pulmonary fibrogenesis by directly targeting miR-344a-5p to regulate map3k11 [21]. Moreover, study has demonstrated that the liver-enriched lncRNA Lfar1 could promote hepatic fibrosis through inducing hepatic stellate cells activation and hepatocytes apoptosis [22]. These findings showed that lncRNAs played an important role in fibrosis progression.

LncRNA KCNQ1OT1, or potassium voltage-gated channel subfamily Q member 1 (Kcnq1) overlapping transcript 1, is an imprinted antisense LncRNA. It is an overlapping transcript of Kcnq1, locating at Kcnq1 loci on chromosome 11p15.5, being exclusively transcribed from the paternal chromosome [23]. Recent reports indicated that lncRNA KCNQ1OT1 was proved to play important roles in a variety of disease. For example, KCNQ1OT1/miR-34c-5p/ALDOA axis could promote osteosarcoma growth via enhancing aerobic glycolysis [24]. Zhang et al. proved that LncRNA KCNQ1OT1 regulates proliferation and cisplatin resistance target miR-211-5p by mediating Ezrin/Fak/Src signaling in tongue cancer [25]. In addition, LncRNA KCNQ1OT1 could affect cell proliferation, apoptosis, and fibrosis through regulating miR-18b-5p/SORBS2 axis in diabetic nephropathy [26]. However, to date, the roles of lncRNA KCNQ1OT1 in renal fibrosis progression was not illuminated.

Hence, our current study was implemented to shed light on the potential role of KCNQ1OT1 in renal fibrosis progression and the underlying mechanism was illuminated by evaluating the relation between KCNQ1OT1 and miR-124-3p. Our findings provide novel prognostic biomarkers for the development of patients with renal diseases.

## Materials and methods

### Animal models

C57BL/6 mice (18 ± 2 g) were purchased from Shanghai Animal Center (Shanghai, People's Republic of China). The experimental protocol was approved by the Ethics Committee for Animal Experimentation of the Fifth Clinical Medical College of Shanxi Medical University. Mice were housed in light/dark cycle for 12 h at 22–24°C and had free to access to food and water. For establishment of UUO model, the ureter was ligated at the proximal hilum of the left kidney, and then the ureter was ligated again away from the hilum of kidney. At the end of the study, the kidneys of each mouse were dissected, and paraffin-embedded renal tissue sections (2 μm) were stained with hematoxylin–eosin (HE), Masson and PAS staining to assess the symptoms of renal fibrosis.

### Cell culture and treatment

Human nephric proximal tubular epithelial cell line HK-2 was purchased from American Tissue Culture Collection (ATCC; Rockville, USA) and grown in DMEM complemented with 10% FBS with 5% CO_2_ in an incubator at 37°C. Cells were starved for 24 h before any stimulation. For establishment of renal fibrosis, HK-2 cells were stimulated with different doses of transforming growth factor β1 (TGF-β1) for different hours and then subjected to further analysis.

### Cell transfection

The sh-KCNQ1OT1, miR-124-3p inhibitor as well as negative control (NC) plasmids were purchased from GenePharma (Shanghai, China). Cell transfection was conducted at final 100 nM concentration using Lipofectamine 2000 (Thermo Fisher Scientific, Waltham, MA, USA). After 48 h, cells were harvested for following experiments.

### Quantitative real-time PCR (qRT-PCR) ananlysis

The total RNA from cells was extracted with Trizol reagent (Invitrogen) and subsequently reversed transcribed into cDNA by utilizing PrimeScript RT reagent Kit (Takara, Ohtsu, Japan). Real-time PCR was carried out with SYBR Premix EX Taq™ II kit (TaKaRa, Dalian, China) on the PCR detection instrument (Opticon CFD-3200; MJ Research, Waltham, MA, USA). U6 and GAPDH were used as internal reference. The quantification of gene relative expression analysis was carried out using the 2^−ΔΔCt^ method [27]. The primer sequences for PCR were displayed as follow: KCNQ1OT1: 5'-GCACTCTGGGTCCTGTTCTC-3' (forward) and 5'-CACTTCCCTGCCTCCTACAC-3' (reverse); miR-124-3p: 5'-TCTTTAAGGCACGCGGTG-3' (forward) and 5'-TATGGTTTTGACGACTGTGTGAT-3' (reverse); GAPDH: 5'-TATGATGATATCAAGAGGGTAGT-3' (forward) and 5'-TGTATCCAAACTCATTGTCATAC-3' (reverse); U6: 5'-CTCGCTTCGGCAGCACA-3' (forward) and 5'-AACGCTTCACGAATTTGCGT-3' (reve-rse).

### Western blot assay

Cells were lysed with RIPA Buffer (cat: 89900; Thermo Fisher Scientific, USA), and the protein concentration was detected with the BCA Kit (cat: P0012S; Beyotime, Beijing, China). The equivalent amount of proteins were segregated on 12% SDS-PAGE and subsequently transferred to PVDF membranes (Millipore, Billerica, MA, USA). Blocking the film with BSA and sealed with 5% skim milk, then the membranes was incubated with the primary antibody against of α-SMA (1:1000; ab5694), Fibronectin (1:1000; ab268021), PCNA (1:1000; ab92552), Ki-67 (1:1000; ab15580) at 4°C overnight, followed by incubation with secondary antibody at room temperature for 1 h. Finally, the bands were measured with enhanced chemiluminescence reagent ECL detection kit (Thermo Fisher Scientifc, Inc.) on ChemiDoc XRS System (Bio-Rad, Hercules, CA, USA). Antibodies mentioned before were supplied by Abcam (Cambridge, UK).

### Cell proliferation assay

Cell viability was assessed using the Cell Counting Kit-8 (CCK-8) assay. In details, cells were plated in 96-well plates at a density of 1 × 10^5^ cells/well and cultivated at 37°C in the presence of 5% CO_2_. At 0, 24, 48, and 72 h post incubation, cells were processed with CCK-8 reagent (Dojindo, Tokyo, Japan), followed by incubation for additional 4 h at 37°C. At last, absorbance was examined at wave length of 450 nm by a microplate reader (BioTek, Winooski, VT, USA).

### Immunofluorescence assay

Transfected cells were cultured for 24 h at 37°C on a Petri dish and fixed in 4% formaldehyde. After PBS washes, cells were penetrated and blocked with 5% BSA for 2 h. Then, the cells were incubated with the primary antibody overnight at 4°C and then incubated with the secondary antibody for 30 min. DAPI (1: 1000) was used to stain the nuclei. The laser scanning fluorescence microscope was used to visualize fluorescence.

### Luciferase reporter assay

The partial sequences of KCNQ1OT1 and the putative binding sites of miR-124-3p were synthesized by Sangon Biotech. The 3ʹUTR of KCNQ1OT1 containing or without binding sites for miR-124-3p were cloned into pGL3 reporter vector (Promega, Madison, WI, USA) to shape KCNQ1OT1-WT or KCNQ1OT1-MUT, respectively. Cells were co-transfected with indicated vectors and miR-124-3p mimic or NC mimic by using Lipofectamine 2000 (Thermo Fisher Scientific, Waltham, MA, USA) according to the product instructions. After 48 h, the luciferase activity was examined using Dual-Luciferase Reporter Assay System (Promega).

### Statistical analysis

All data were shown as means ± SD via three parallel experiments. Statistical analysis was conducted by employment of SPSS 21.0 software. Student's t test and one-way ANOVA were applied for comparison between two or more groups. Differences were defined as statistically significant when P < 0.05.

## Results

### Silencing of KCNQ1OT1 attenuated the symptom of renal fibrosis in vivo

**Figure 1.**
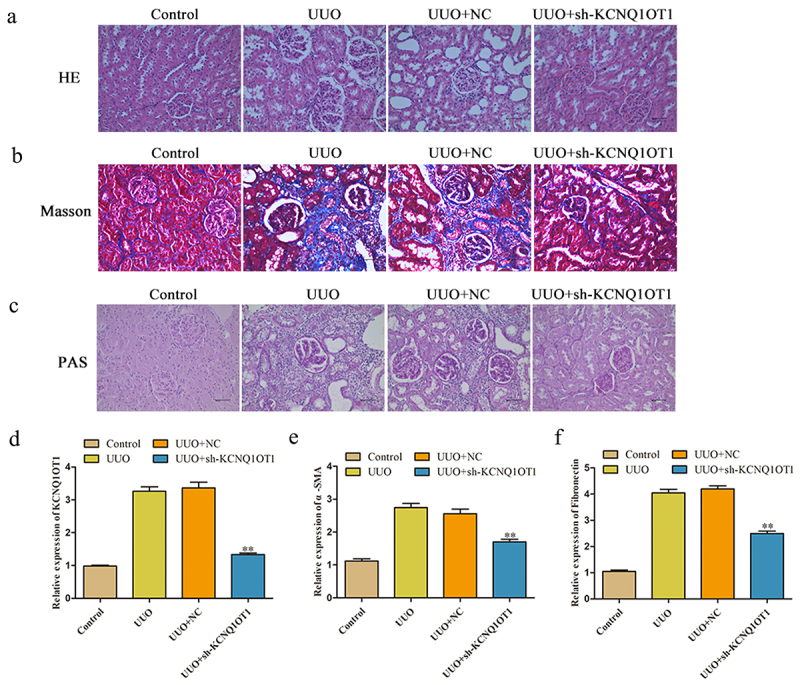
Silencing of KCNQ1OT1 attenuated the symptom of renal fibrosis in vivo. (a-c) H&E, Masson and PAS staining of mice kidney tissue were detected. (d) The expression of KCNQ1OT1 in kidney tissues of mice was analyzed by qRT-PCR assay. (e) The expression level of α-SMA and Fibronectin of kidney tissues was measured. **P < 0.01 *vs*. UUO.

To gain insights into the potential effect of KCNQ1OT1 in renal fibrosis, H&E, Masson and PAS staining were performed to evaluate the pathological changes in the kidney of mice. As demonstrated in Figure 1(a), the kidney tissues showed infiltration of inflammatory cells and cellular degeneration in UUO model, KCNQ1OT1 shRNA notably inhibited UUO induced renal fibrosis in mice. As depicted in Figure 1(b,c), UUO group exhibited fibrosis with massive blue-stained fiber, while the symptom of renal fibrosis was obviously decreased by knockdown of KCNQ1OT1 expression. In addition, the expression of KCNQ1OT1 in kidney tissues of mice was notably increased by UUO, which was partially reversed in the presence of kidney shRNA (Figure 1(d)). Finally, UUO-induced increase of α-SMA and Fibronectin level in mice and was obviously inhibited by KCNQ1OT1 knockdown (Figure 1(e,f)).

### The KCNQ1OT1 expression was increased in TGF-β-induced HK-2 cells

**Figure 2.**
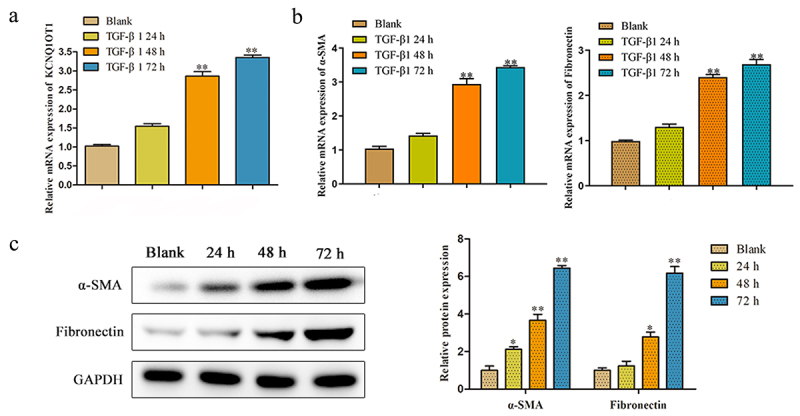
The KCNQ1OT1 expression was decreased in TGF-β-induced HK-2 cells. (a) The KCNQ1OT1 expression in TGF-β-induced HK-2 cells from 0 h to72 h. (b, c) The mRNA and protein expressions of α-SMA and Fibronectin with treatment with TGF-β (10 ng/ml) was detected by RT-PCR and western blot analyses. All results were presented as mean ± SD from at least three independent experiments. *P < 0.05, **P < 0.01 *vs*. Blank.

We investigated the role of KCNQ1OT1 in renal fibrosis using HK-2 cells in vitro. After incubation with the pro-fibrotic cytokine TGF-β1, the expression of KCNQ1OT1 was markedly increased at 48 h and 72 h (Figure 2(a)). Moreover, the main components of ECM including α-SMA and Fibronectin were significantly induced in HK-2 cells after TGF-β stimulation (Figure 2(b,c)). These results potentially indicated that KCNQ1OT1 plays a protective role in attenuating renal fibrosis.

### Knockdown of KCNQ1OT1 inhibited cell proliferation of TGF-β1-induced HK-2 cells

**Figure 3.**
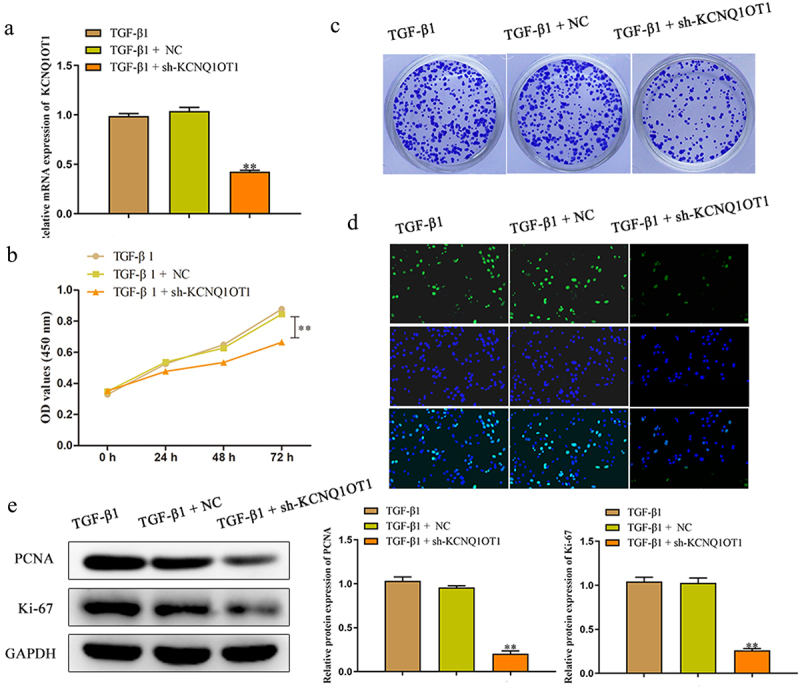
Downregulation of KCNQ1OT1 inhibited cell proliferation of TGF-β1-induced HK-2 cells. (a) qRT-PCR assay was performed to detect the transfected efficiency after transfection for 48 h of HK-2 cells which stimulated with TGF-β1 for 48 h. (b-d) CCK-8, colony formation and EdU assays were used to assess the proliferation ability of HK-2 cells which stimulated with TGF-β1 for 48 h. (e) The expression levels of PCNA and Ki-67 were analyzed by western blot assay. All results were presented as mean ± SD from at least three independent experiments. **P < 0.01 *vs*. TGF-β1.

In order to investigate the effects of KCNQ1OT1 on renal fibrosis in vitro, HK-2 cells were transfected with sh-KCNQ1OT1 and negative control, and qRT-PCR assay was performed to detect the transfected efficiency. The results certified that sh-KCNQ1OT1 led to suppression of KCNQ1OT1 in TGF-β1-induced HK-2 cells compared to the sh-NC group (Figure 3(a)). Moreover, we explore the effect of KCNQ1OT1 on the viability of TGF-β1-induced HK-2 cells by the CCK-8 assay. The data of Figure 3(b) demonstrated that down-expression of KCNQ1OT1 remarkably suppressed the viability of HK-2 cells, when compared with sh-NC group. Subsequently, the role of KCNQ1OT1 in the proliferation of TGF-β1-induced HK-2 cells was determined by colony formation assay. The results verified that transfection with sh-KCNQ1OT1 significantly suppressed the proliferation of TGF-β1-induced HK-2 cells (Figure 3(c)). The data of EdU assay revealed that the EdU-positive cells in HK-2 cells transfected with sh-KCNQ1OT1 were obviously reduced compared to sh-NC group (Figure 3(d)). In addition, western blot assay was carried out to assess the effects of KCNQ1OT1 on the cell proliferation-related proteins. Down-regulating KCNQ1OT1 dramatically suppressed the expression levels of Ki-67 and PCNA in TGF-β1-induced HK-2 cells (Figure 3(e)).

### Suppression of KCNQ1OT1 inhibits the expression of α-SMA and Fibronectin in TGF-β1-induced HK-2 cells

**Figure 4.**
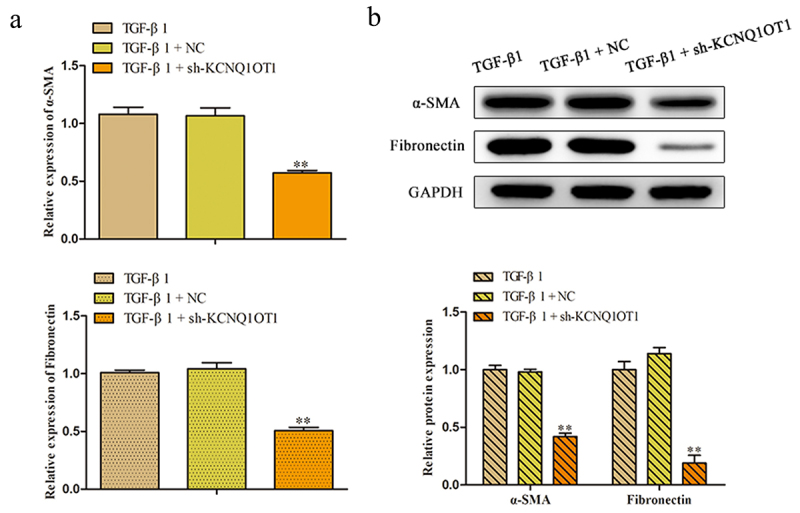
Silencing of KCNQ1OT1 inhibits the expression of α-SMA and Fibronectin in TGF-β1-induced HK-2 cells. (a, b) The relative mRNA and protein expression of α-SMA and Fibronectin were analyzed by using qRT-PCR and western blote analyses. **P < 0.01 *vs*. TGF-β1.

To further explore the roles of KCNQ1OT1 in the renal fibrosis, the qRT-PCR was applied to detect the relative expression of α-SMA, a marker of myofibroblasts, and Fibronectin, the major components of ECM. The results of Figure 4(a) shown that the expression level of α-SMA and fibronectin were augmented caused by TGF-β1 treatment and decreased by transfecting with sh-KCNQ1OT1 in HK-2 cells. Meanwhile, western blot analysis manifested that silenced of KCNQ1OT1 aggravated the diminution of protein levels of α-SMA and Fibronectin that increased by TGF-β1 administration (Figure 4(b)).

### MiR-124-3p is a direct target of KCNQ1OT1 in renal fibrosis

**Figure 5.**
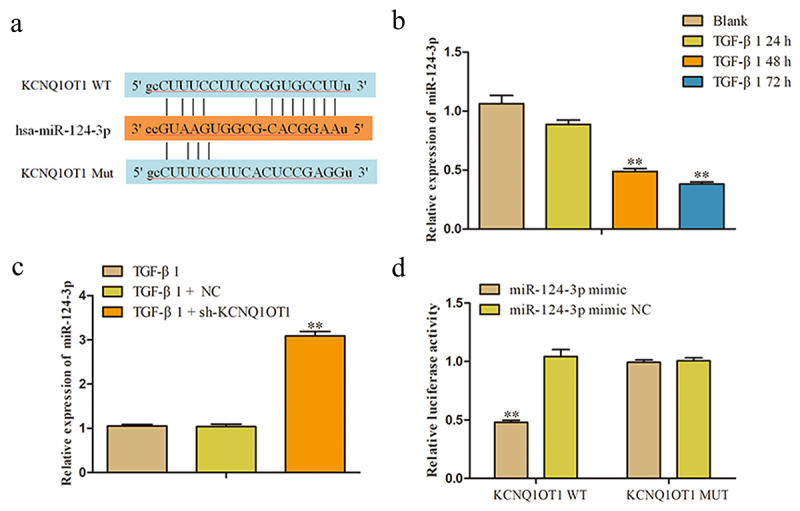
MiR-124-3p was a downstream target of KCNQ1OT1. (a) The binding site between KCNQ1OT1 and miR-124-3p were shown. (b, c) The expression of miR-124-3p was detected by RT-PCR. **P < 0.01 *vs*. Blank or TGF-β1. (d) Luciferase reporter assay was performed to verify the targeting relationship between KCNQ1OT1 and miR-124-3p. All results were presented as mean ± SD from at least three independent experiments. **P < 0.01 *vs*. Blank, TGF-β1 or miR-124-3p mimic NC.

It is well known that lncRNAs exhibit multiple biological functions by sponging various miRNAs. In order to study on the possible target genes of KCNQ1OT1 involved in the occurrence and progression of renal fibrosis and assessed the KCNQ1OT1-associated miRNAs, we used bioinformatics analysis (Starbase, DIANA, and ENCORI) to search for genes that were directly regulated by KCNQ1OT1. Among all the targets, miR-124-3p was chosen for further study, since miR-124-3p functions as an important regulator controlling the occurrence and development of various fibrosis [28–30]. The binding site between KCNQ1OT1 and miR-124-3p were as shown in Figure 5(a). In addition, the miR-124-3p expression in TGF-β-induced HK-2 cells was analyzed from 0 h to 72 h and results indicated that the expression of miR-124-3p was significantly decreased at 48 h and 72 h (Figure 5(b)). Furthermore, we found that miR-124-3p was negatively regulated by KCNQ1OT1. The expression level of miR-124-3p was markedly increased in sh-KCNQ1OT1 group compared with pcDNA3.1 group (Figure 5(c)).

To further validate whether KCNQ1OT1 directly binding to miR-124-3p, luciferase reporter assay was performed. The luciferase reporter plasmids were contained with the 3' UTR of KCNQ1OT1 wild type (WT) or mutant (Mut). As shown in Figure 5(d), the luciferase activity of KCNQ1OT1-WT was significantly decreased when co-transfected with miR-124-3p mimic, while no significant alterations were observed mutation at miR-124-3p binding site in the 3' UTR of KCNQ1OT1.

### KCNQ1OT1 inhibits renal fibrosis through suppression of miR-124-3p expression

**Figure 6.**
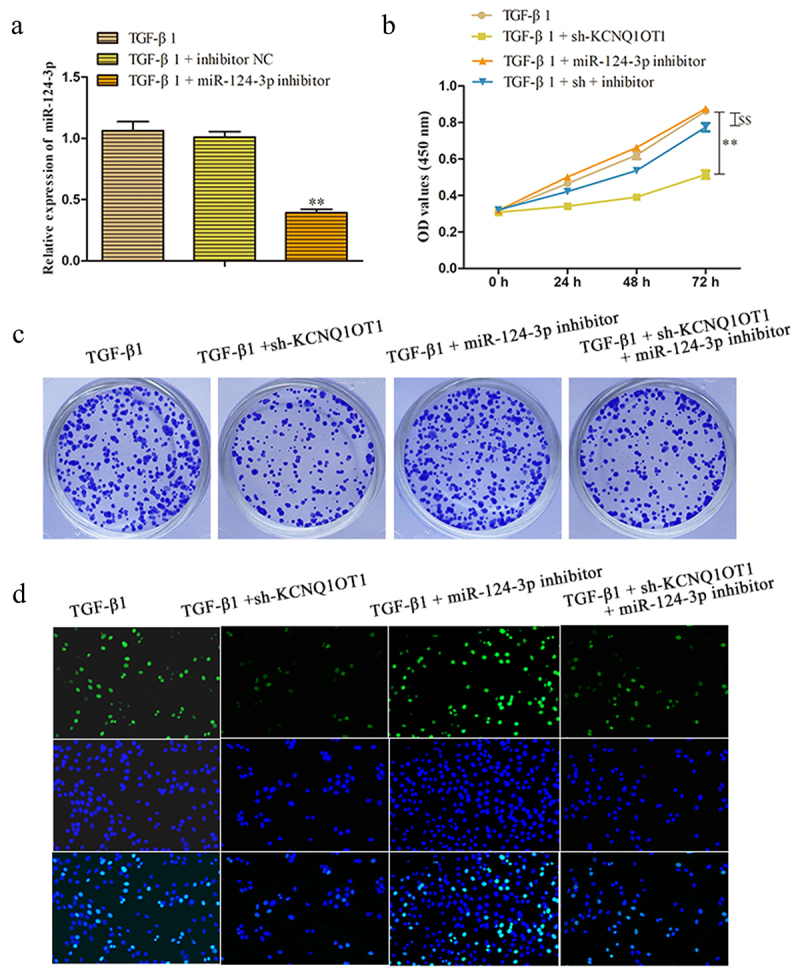
KCNQ1OT1 knockdown inhibits renal fibrosis through suppression of miR-124-3p expression. (a) The expression levels of miR-124-3p were measured to test the transfected efficiency. (b-d) Cell proliferation was measured by CCK-8, colony formation and EdU assays. All results were presented as mean ± SD from at least three independent experiments. **P < 0.01 *vs*. TGF-β1. ^$$^*P* < 0.01 *vs*. TGF-β1 + sh-KCNQ1OT1.

**Figure 7.**
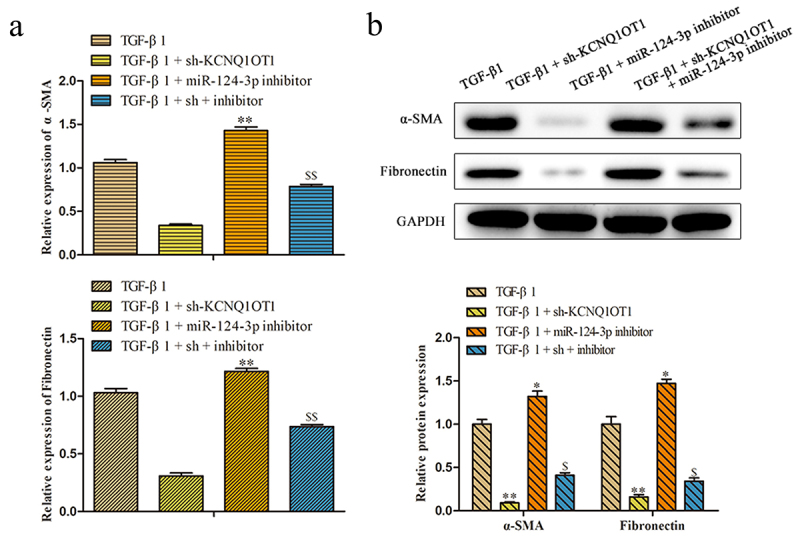
KCNQ1OT1 regulated renal fibrosis-associated markers through suppression of miR-124-3p expression. (a, b) qRT-PCR and western blot assays were applied to analyzed the mRNA and protein expression levels of α-SMA and Fibronectins. All results were presented as mean ± SD from at least three independent experiments. *P < 0.01, **P < 0.01 *vs*. TGF-β1. ^$^*P* < 0.05, ^$$^*P* < 0.01 *vs*. TGF-β1 + sh-KCNQ1OT1.

To further investigate the role of KCNQ1OT1/miR-124-3p in the regulation of renal fibrosis, we artificially down-expressed miR-124-3p in HK-2 cells. RT-PCR assay disclosed that the expression of miR-124-3p was successfully decreased by transfected with miR-124-3p inhibitor (Figure 6(a)). To confirm whether the effect of KCNQ1OT1 on renal fibrosis was mediated by miR-124-3p, miR-124-3p inhibitor was co-transfected with KCNQ1OT1 knockdown in HK-2 cells (TGF-β1 + sh-KCNQ1OT1 + miR-124-3p inhibitor). Proliferative capacity was determined by CCK-8 and colony formation assays. The data displayed that miR-124-3p inhibitor abolished the repressive effects of KCNQ1OT1 downexpression on TGF-β1-induced HK-2 cell viability (Figure 6(b,c)). Consistently, the numbers of EdU-positive cells were increased correspondingly to the sh-KCNQ1OT1 + miR-124-3p inhibitor -transfected HK-2 cells compared with those in sh-KCNQ1OT1 group (Figure 6(d)). Additionally, qRT-PCR and western blot assays illustrated that miR-124-3p inhibitor abolished the inhibitory effects of KCNQ1OT1 suppression on the mRNA and protein expression levels of α-SMA and Fibronectin in TGF-β1-induced HK-2 cells (Figure 7).

## Discussion

Renal fibrosis is increasingly becoming a major public health issue and considered as the common final stage of progressive renal disease [31]. The pathogenesis of renal fibrosis is a progressive process that reduces the capacity for tissue repair and ultimately leads to end-stage kidney failure [32,33].

Mounting evidence has emphasized that aberrant expression levels of lncRNAs and miRNAs were contributed to the kidney diseases including renal fibrosis [34,35]. Studies have demonstrated that ectopic the expression of lncRNA-SARCC could decrease RCC cells resistance to Sunitinib [36]. Wang et al. proved that knockdown of lncRNA GAS5 contributed to anti-fibrosis by competitively binding miR-96-5p [37]. Moreover, lncRNA NR_038323 is also revealed to suppress HG-induced renal fibrosis through the miR-324-3p/DUSP1 axis [38]. Besides, lncRNA-LET serves a tumor suppressive role by regulating miR-373-3p in RCC [39].

As a typical multifunctional lncRNA, KCNQ1OT1 is reported widely expressed in various diseases and plays a crucial role in multiple molecular and cellular processes. For instance, silencing lncRNA KCNQ1OT1 could alleviate pyroptosis and fibrosis in diabetic cardiomyopathy [40]. Li et al. also found that lncRNA KCNQ1OT1 enhances the chemoresistance of oxaliplatin by targeting the miR-34a/ATG4B pathway in colon cancer [41]. Moreover, LncRNA KCNQ1OT1 has been proved could regulate tongue cancer cell proliferation and cisplatin resistance via miR-211-5p mediated Ezrin/Fak/Src signaling [25]. However, explorations about the exact role of KCNQ1OT1 in the progression of renal fibrosis are scanty. Based on the literature, we hypothesize that KCNQ1OT1 may play regulatory function in the progression of renal fibrosis.

TGF-β1 is an important factor responsible for renal fibrosis. Herein, we used HK-2 cells induced with TGF-β1 to establish the cellular renal fibrosis model in vitro. In the present study, we concentrated on the potential function of KCNQ1OT1 and further investigated its latent molecular mechanism in the renal fibrosis progression. We found that the expression level of miR-124-3p was overtly down-regulated in TGF-β-induced HK-2 cells.

Mounting evidence has emphasized that the main pathological change of renal fibrosis is the deposition of ECM and TGF-β1-activated lung fibroblasts conceivably overlap with α-SMA positive myofibroblasts, which is responsible for producing ECM components, such as Fibronectin [42,43]. Next, we performed loss-of-function experiments identified that silencing of KCNQ1OT1 expression inhibits the proliferation and EMC deposition including α-SMA and Fibronectin in TGF-β1-induced HK-2 cells.

MiRNAs are group of small, non-coding RNAs with the nucleotides about 22 and are identified as a negative regulator. Studies have shown that miRNA have become inhibitory or carcinogenic in tumorigenesis and the expression of lncRNAs can regulate the activities of miRNAs [44,45]. LncRNAs often function as competing endogenous RNAs binding to miRNA, then exert their function. Hence, we speculated that KCNQ1OT1 effected renal fibrosis process through sponging the specific miRNA. We used multiple-bioinformatic analyses to search for genes that were directly regulated by KCNQ1OT1. In this study, we found that miR-124-3p was a target miRNA of KCNQ1OT1. Accordingly, the expression level of miR-124-3p was negatively regulated with the KCNQ1OT1. In addition, luciferase assays revealed that miR-124-3p could bind to KCNQ1OT1 3' UTR and decrease its luciferase activity in the HK-2 cells.

Moreover, to investigate the relationship between KCNQ1OT1 and miR-124-3p in the progress of renal fibrosis, rescue experiment was performed. It was revealed that co-transfection with sh-KCNQ1OT1 and miR-124-3p inhibitor attenuated the inhibitory effect of sh-KCNQ1OT1 on the proliferation and the expression levels of α-SMA and Fibronectin of HK-2 cells, suggesting that KCNQ1OT1 interacted with miR-124-3p and inhibited miR-124-3p expression, thus regulating the occurrence and development of renal fibrosis. These results provided evidence that KCNQ1OT1 may function as a protector in renal fibrosis progression through targeting miR-124-3p.

## Conclusion

Our study uncovered the first clue that KCNQ1OT1 knockdown plays an anti-fibrotic effect through the suppression of cell proliferation and down-regulation of α-SMA and Fibronectin expression by directly targeting miR-124-3p. On the whole, these findings provide a deep insight into the mechanisms underlying renal fibrosis, which developed a new therapeutic target for clinical prevention from devastating processes of renal fibrosis.

## Supplementary Material

Supplemental Material

## Data Availability

The datasets used and/or analyzed for the current study are available from the corresponding author upon reasonable request.
