# Supplementary material for: Silencing of LncRNA KCNQ1OT1 confers an inhibitory effect on renal fibrosis through repressing miR-124-3p activity
Source: Bioengineered. 2022 Apr 21;13(4):10399–411. doi: 10.1080/21655979.2022.2056816 (PMC9161840; doi:10.1080/21655979.2022.2056816)
Supplement: Supplemental Material [file KBIE_A_2056816_SM9779.zip › supplementary/ethical.pdf]

山西医科大学第五临床医学院 科研伦理审查申请表

<table>
<tr><td>项目依托单位</td><td>山西医科大学第五临床医学院</td><td colspan="2">序号（由委员会统一填写）</td><td></td></tr>
<tr><td>项目名称</td><td colspan="2">LncRNAs 对肾纤维化的影响及作用机制研究</td><td>项目起止时间</td><td>2020/06-2023/06</td></tr>
<tr><td>项目来源</td><td colspan="4">☐拟申请课题或资金来源：＿＿＿＿＿＿＿＿ ☑自发项目
☐已获横向项目立项课题：＿＿＿＿＿＿＿＿</td></tr>
<tr><td>项目类别</td><td colspan="4">☑干预性研究　☐非干预性研究（☐前瞻性研究　☐回顾性研究 ）</td></tr>
</table>

申请人（项目负责人）简要信息

姓名	郝建	性别	男	移动电话		电子邮箱	rejoicejian@163.com
学历	MM	科室	肾内科	目前主要研究方向			

研究内容摘要

<table>
<tr><td>研究背景和目的</td><td>慢性肾脏病 (Chronic kidney disease, CKD) 是各种肾脏病的总称，已逐渐发展成一种全球性疾病。在过去 30 年中，CKD 的患病率和发病率上升了近 90%。众所周知，在 CKD 中，纤维化旨在保护组织免受损伤，是一种主动的生物愈合反应。然而，当肾脏受到慢性且持续的损伤后，致使组织修复/再生能力异常，引发肾纤维化，并最终导致肾衰竭。因此，我们希望可以发现针对肾脏纤维化的新疗法，以减缓这些人的疾病进展速度，减轻患者的经济负担。
在本课题中，我们拟深入研究 LncRNAs 在抗肾脏纤维化的作用机制，解析其发挥作用的分子机制，为探索新的肾纤维化的分子靶点提供理论基础。</td></tr>
<tr><td>研究处理因素</td><td>小鼠肾纤维化模型（UUO）建立：在左肾近端门部结扎输尿管，然后再次从肾门处结扎输尿管。</td></tr>
</table>

受试者选择	C57BL/6 小鼠
对照设置、干预措施及观察指标	将小鼠置于 22 - 24°C 的光/暗循环中 12 小时，并可自由获取食物和水。研究结束时，对每只小鼠的肾脏进行解剖，并对石蜡包埋的肾组织切片（2μm）。<br>假手术组小鼠注射等量 PBS。动物水平通过 UUO 小鼠模型敲低 KCNQ1OT1，苏木精-伊红（HE）、Masson 和 PAS 染色分析肾脏组病理结构变化、肾纤维化相关指标包括α-SMA 和纤维粘连蛋白（Fibronectin）表达水平评估小鼠的肾损伤情况。
风险/收益分析	/
特殊审查条件（可多选）	□研究涉及生物标本的出口　□研究涉及数据的跨国传送　□研究涉及生物样本库的建立　□利用干细胞进行研究　□研究涉及常规医疗/体检以外的生物样本采集、侵入性检查、放射性检查　□研究涉及弱势群体（□儿童/未成年人　□认知障碍或因健康状况而没有能力做出知情同意的成人　□疾病终末期病人）

申请人（项目负责人）承诺：

以上所填内容均属实，如获批准，我将严格按照提供的方案进行研究，并遵守医院科研伦理委员会的相关规定。

申请人（项目负责人）签字：[illegible] 日期：2020. 5. 8 所在科室（盖章）：

科研伦理委员会审批意见：

主任委员（签章）： 日期：2020. 5. 9 山西医科大学第五临床医学院科研伦理委员会（盖章）

山西医科大学第五临床医学院 伦理委员会
