# Supplementary figures and images for: Silencing of LncRNA KCNQ1OT1 confers an inhibitory effect on renal fibrosis through repressing miR-124-3p activity
Source: Bioengineered. 2022 Apr 21;13(4):10399–411. doi: 10.1080/21655979.2022.2056816 (PMC9161840; doi:10.1080/21655979.2022.2056816)

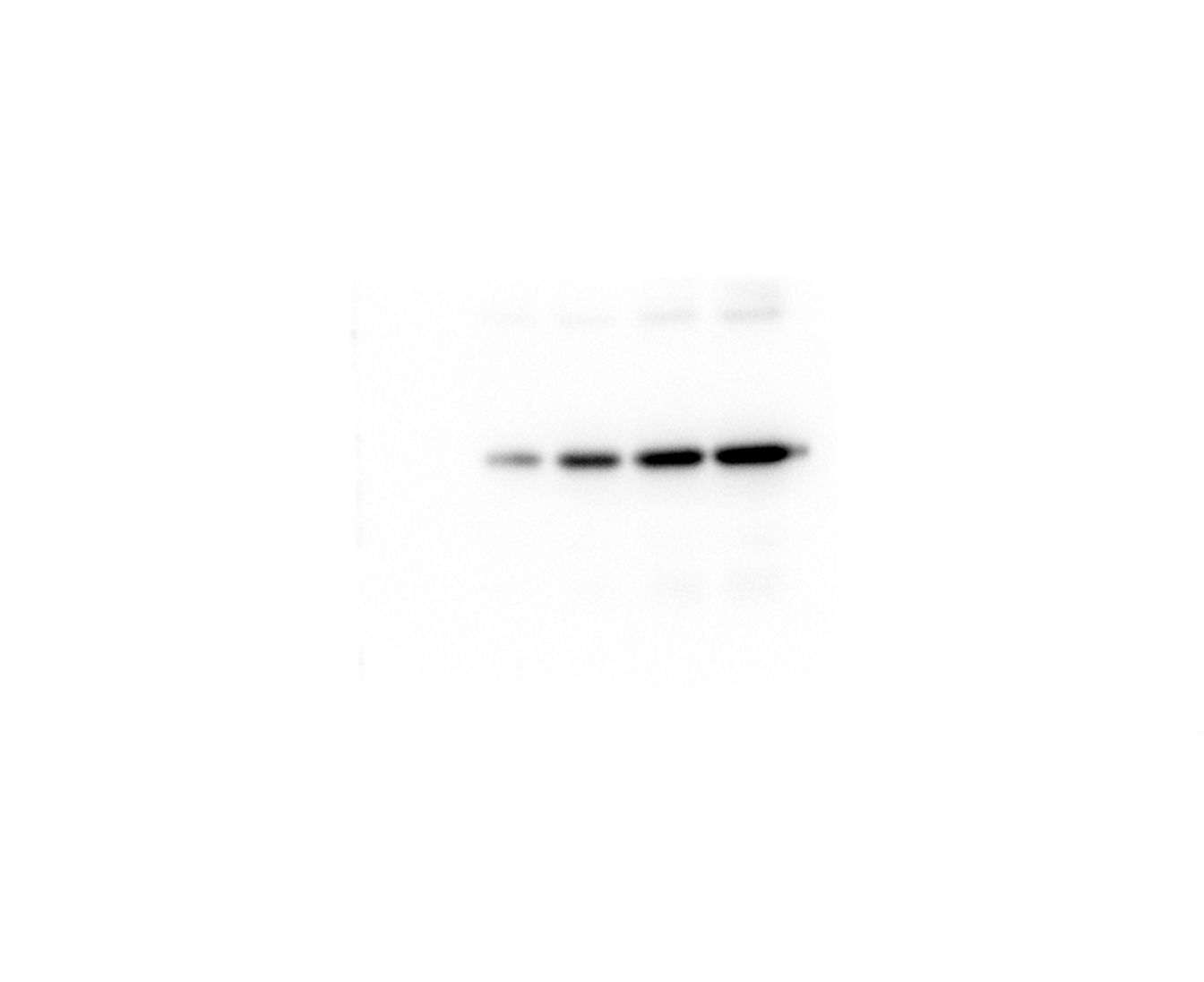

Supplement: Supplemental Material [file KBIE_A_2056816_SM9779.zip › supplementary/2C a SMA.Tif]

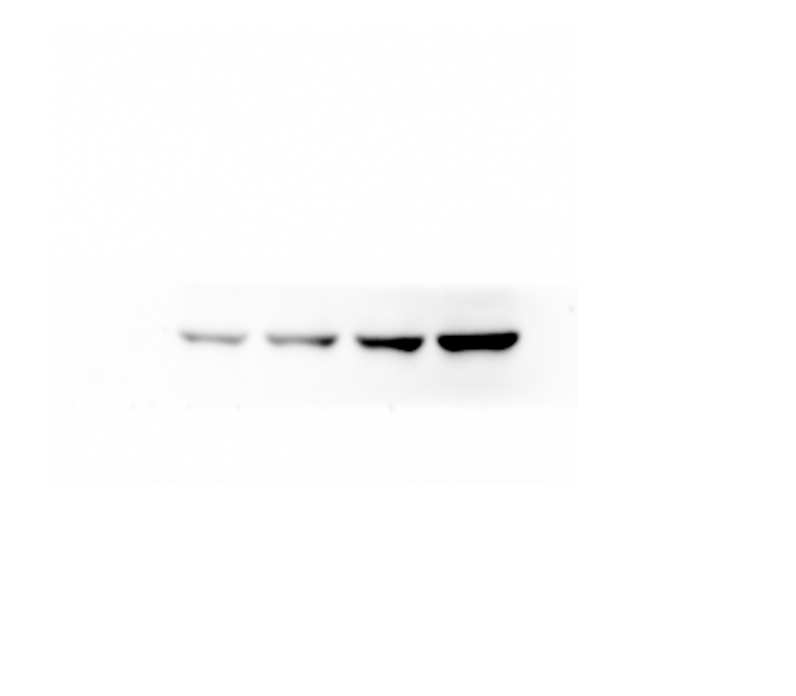

Supplement: Supplemental Material [file KBIE_A_2056816_SM9779.zip › supplementary/2C Fibronectin.tif]

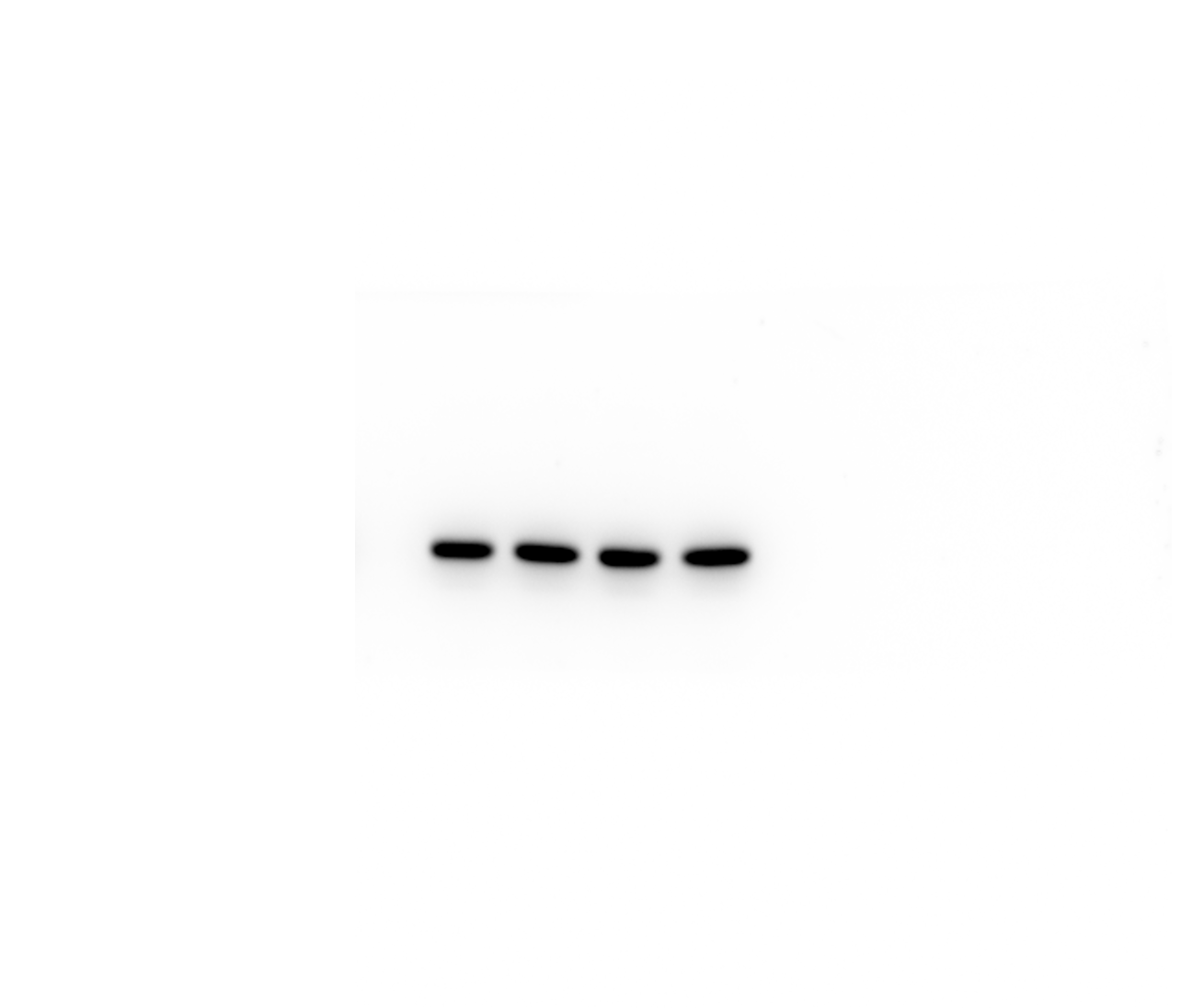

Supplement: Supplemental Material [file KBIE_A_2056816_SM9779.zip › supplementary/2C GAPDH.tif]

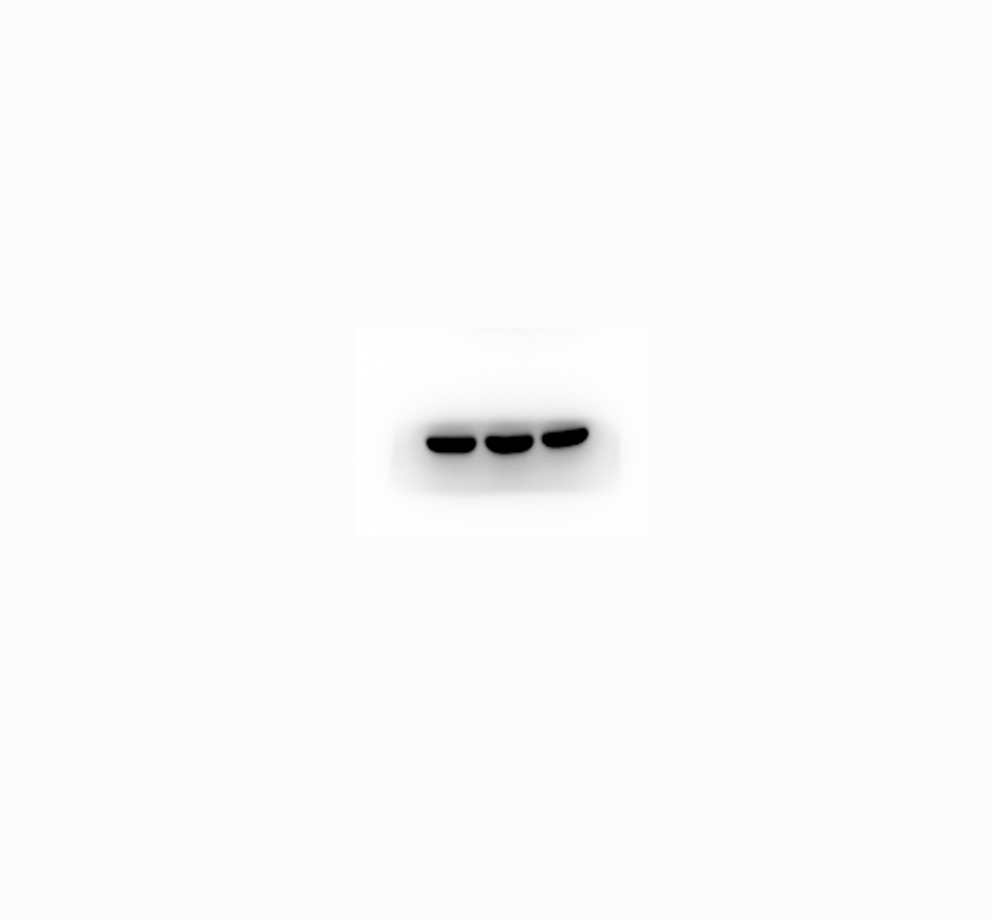

Supplement: Supplemental Material [file KBIE_A_2056816_SM9779.zip › supplementary/3E GAPDH.tif]

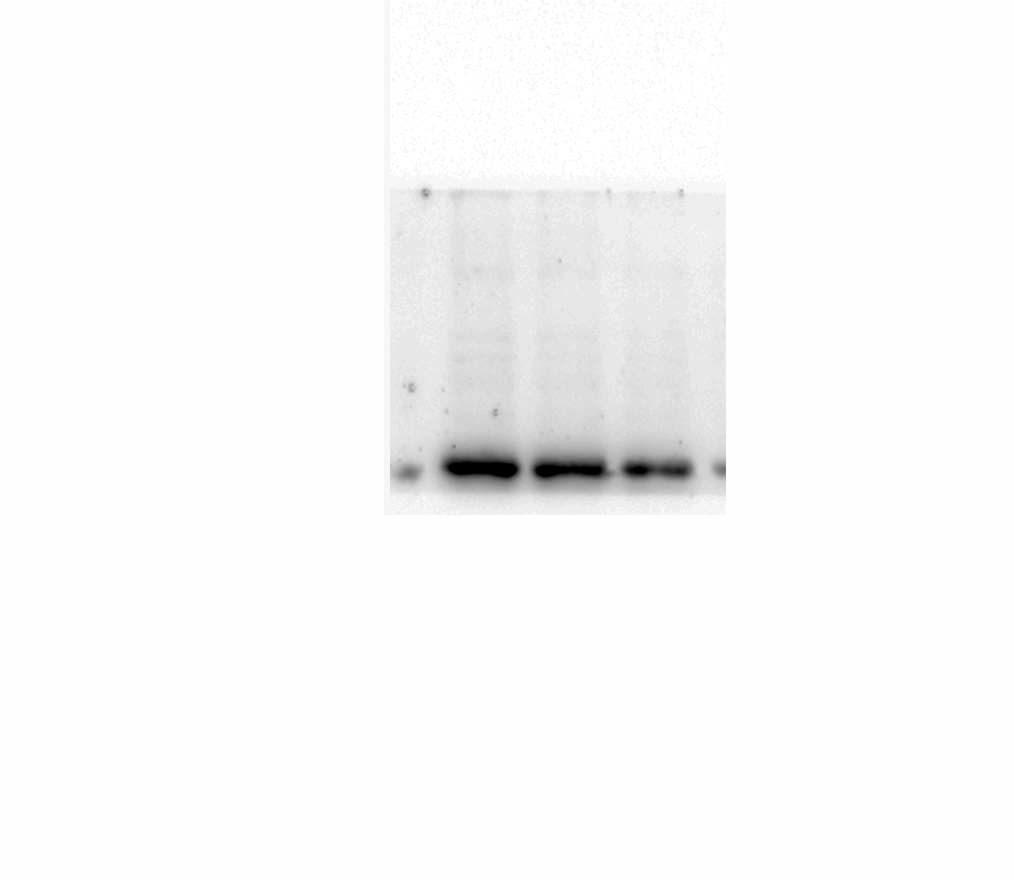

Supplement: Supplemental Material [file KBIE_A_2056816_SM9779.zip › supplementary/3E Ki 67.tif]

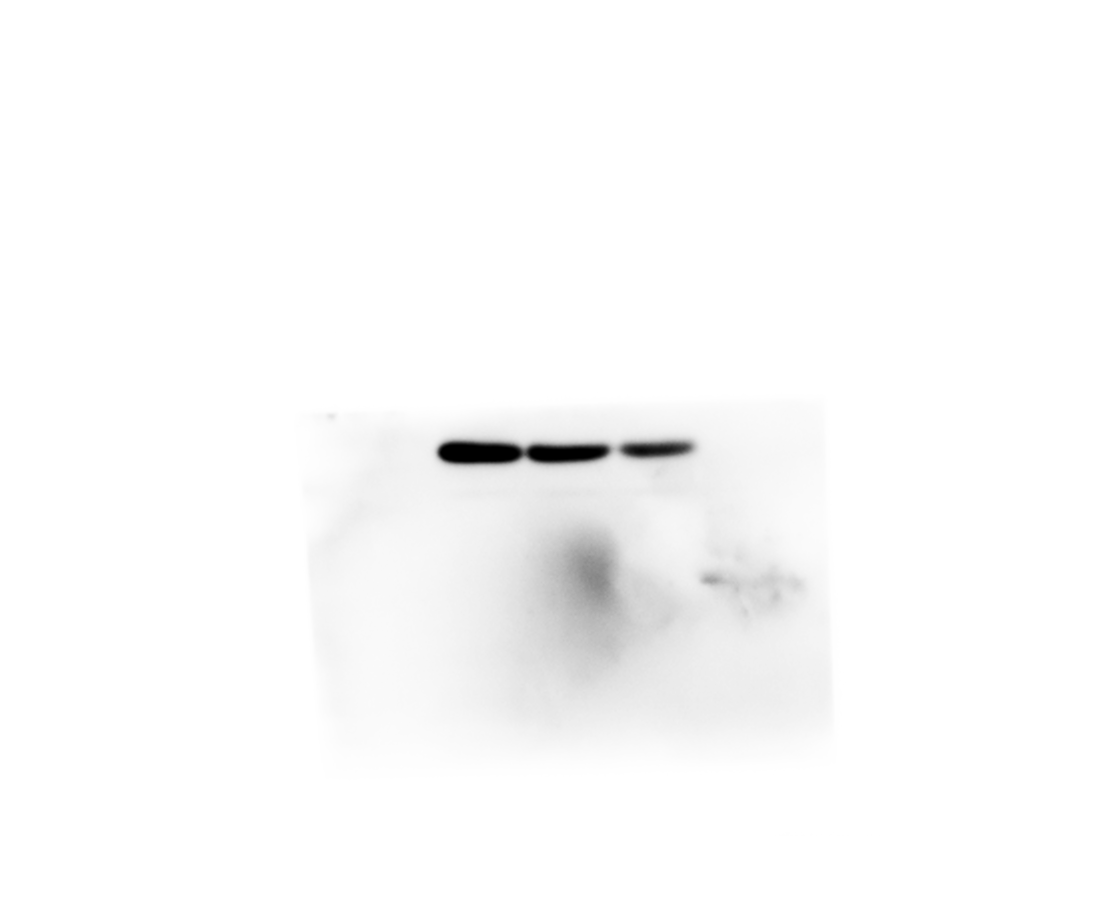

Supplement: Supplemental Material [file KBIE_A_2056816_SM9779.zip › supplementary/3E PCNA.tif]

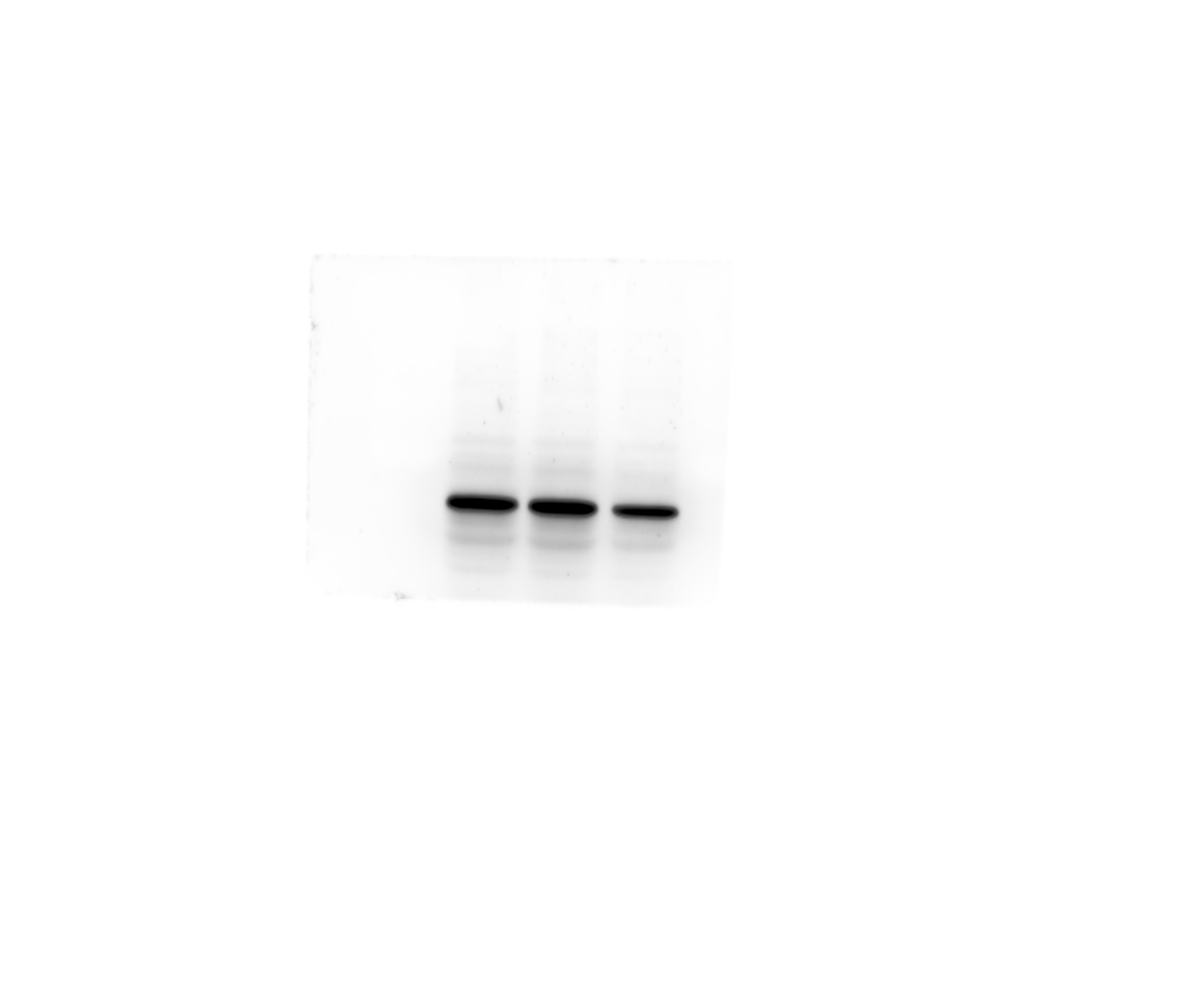

Supplement: Supplemental Material [file KBIE_A_2056816_SM9779.zip › supplementary/4B a SMA.Tif]

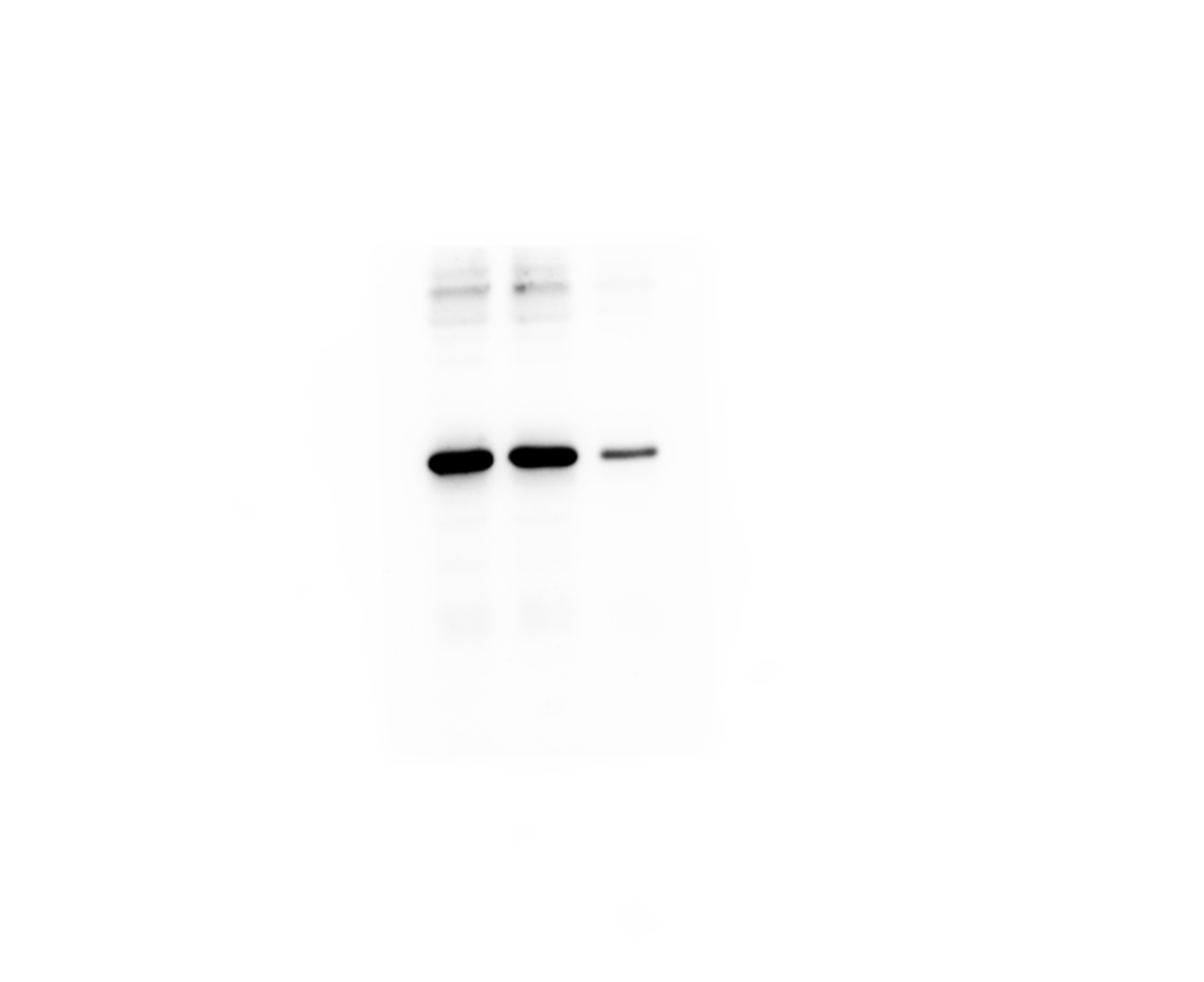

Supplement: Supplemental Material [file KBIE_A_2056816_SM9779.zip › supplementary/4B Fibronectin.Tif]

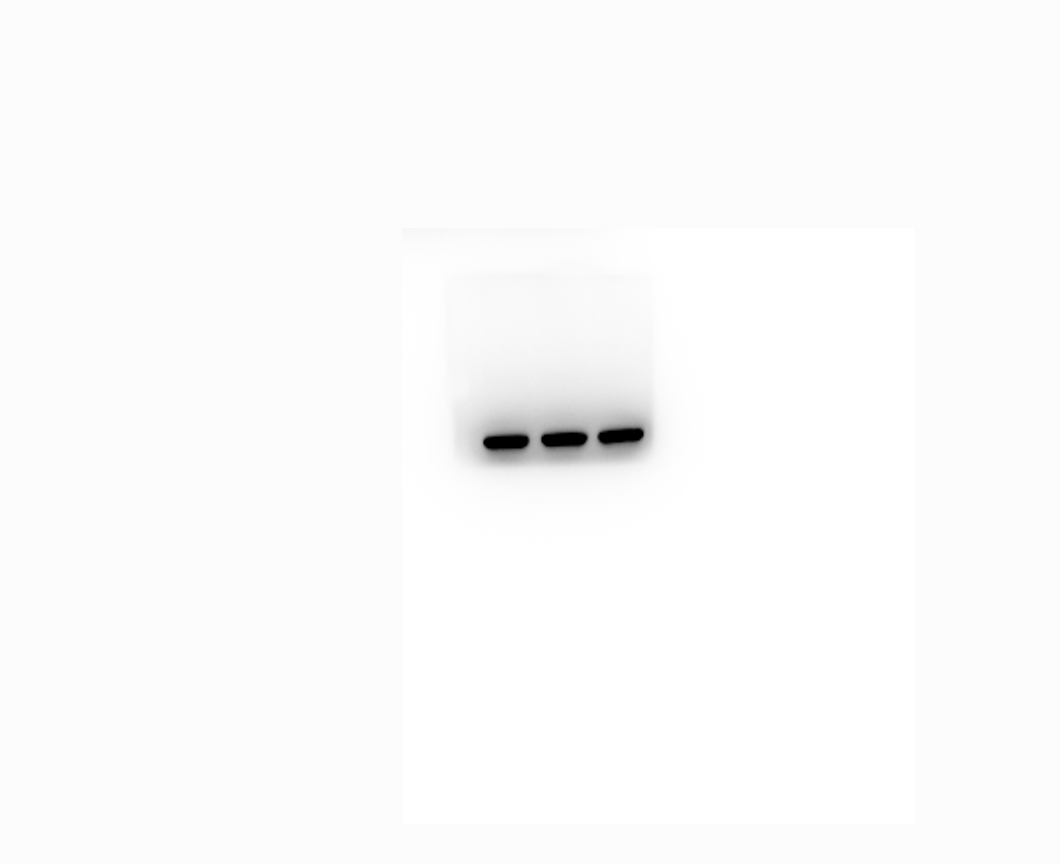

Supplement: Supplemental Material [file KBIE_A_2056816_SM9779.zip › supplementary/4B GAPDH.tif]

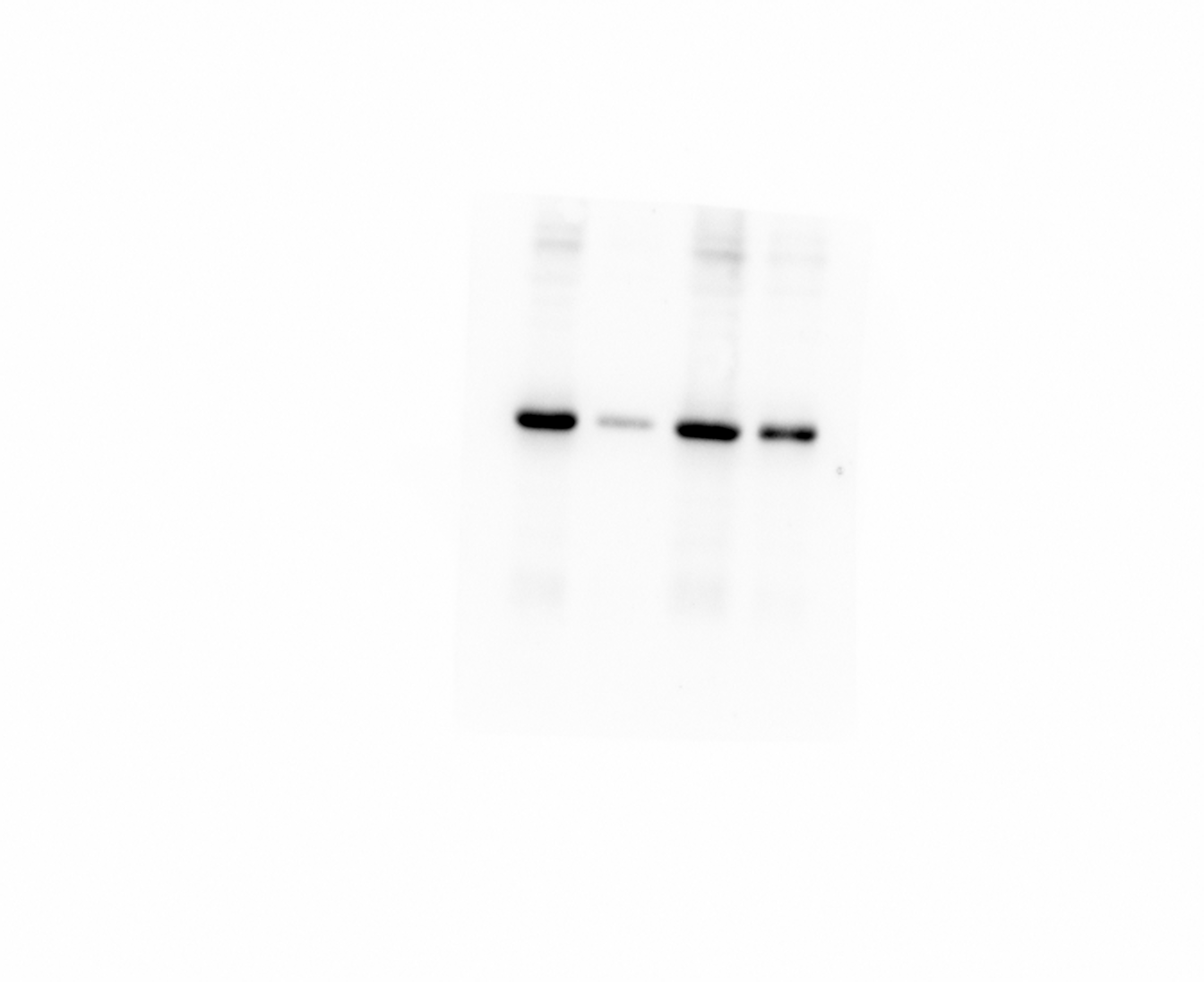

Supplement: Supplemental Material [file KBIE_A_2056816_SM9779.zip › supplementary/7B a SMA.Tif]

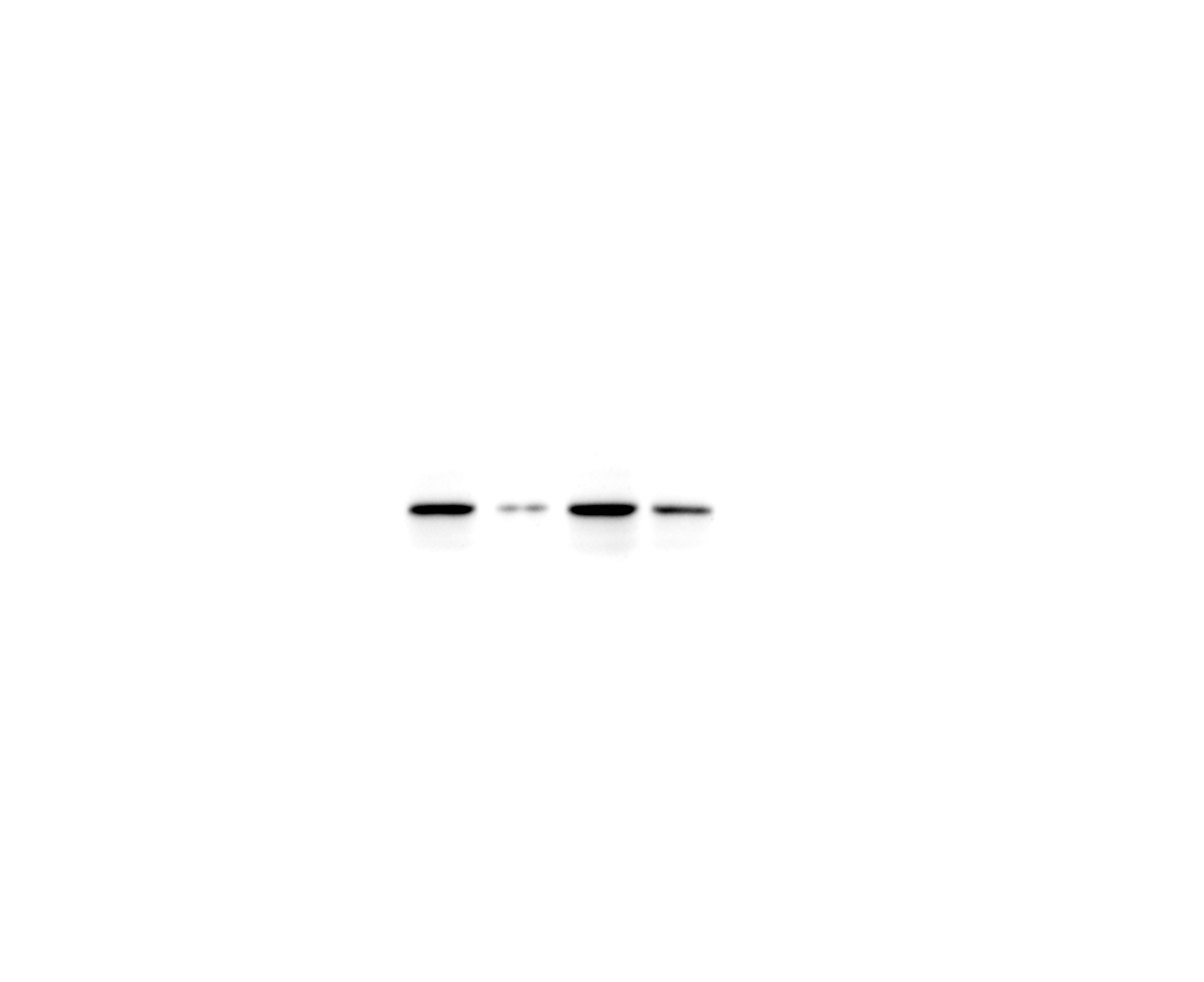

Supplement: Supplemental Material [file KBIE_A_2056816_SM9779.zip › supplementary/7B Fibronectin.Tif]

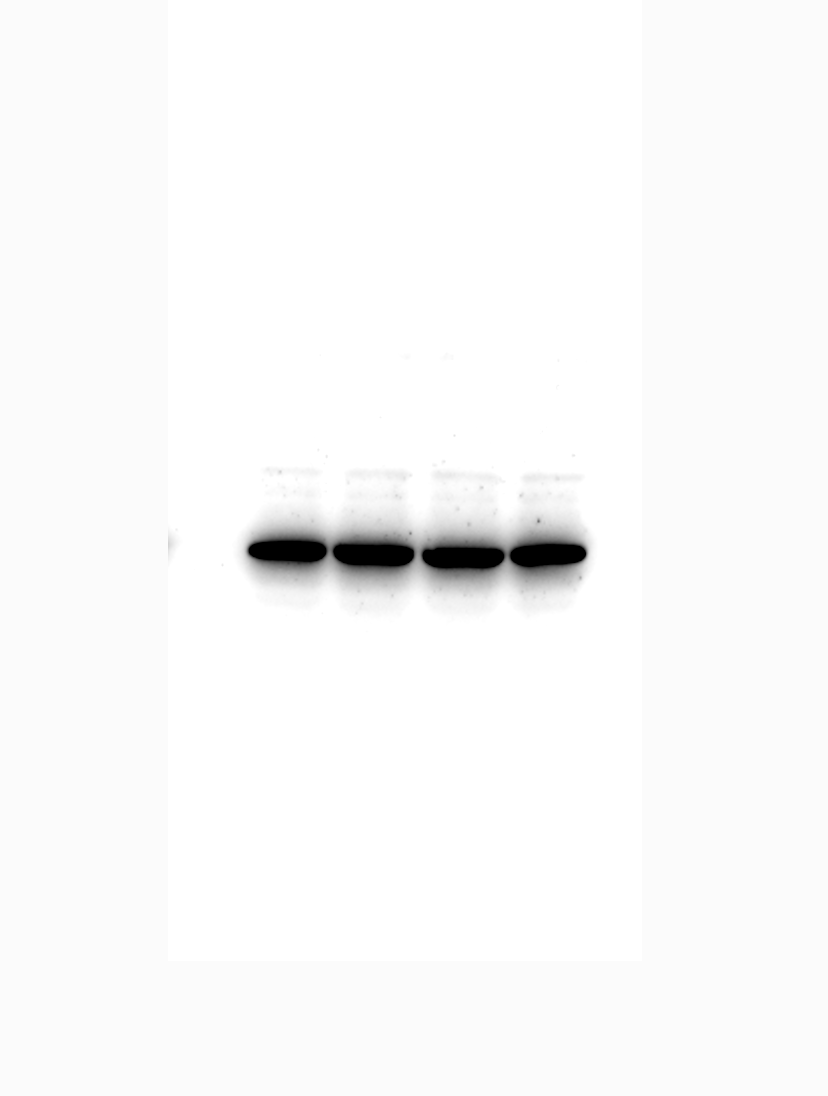

Supplement: Supplemental Material [file KBIE_A_2056816_SM9779.zip › supplementary/7B GAPDH.tif]
